# Urine E-cadherin: A Marker for Early Detection of Kidney Injury in Diabetic Patients

**DOI:** 10.3390/jcm9030639

**Published:** 2020-02-27

**Authors:** Michael Koziolek, Gerhard A. Mueller, Gry H. Dihazi, Klaus Jung, Constanze Altubar, Manuel Wallbach, Ivana Markovic, Dirk Raddatz, Olaf Jahn, Hülya Karaköse, Christof Lenz, Henning Urlaub, Abdelhi Dihazi, Abdellatif El Meziane, Hassan Dihazi

**Affiliations:** 1 Clinic for Nephrology and Rheumatology, University Medical Center Göttingen 1, 37075 Göttingen, Germany; mkoziolek@med.uni-goettingen.de (M.K.); constanze@schweingruber.eu (C.A.); manuel.wallbach@med.uni-goettingen.de (M.W.); huelya.karakoese@stud.uni-goettingen.de (H.K.); 2 Institute for Clinical Chemistry/UMG-Laboratories, University Medical Center Göttingen, 37075 Göttingen, Germany; gryhelene.dihazi@med.uni-goettingen.de (G.H.D.); ivana.markovic@med.uni-goettingen.de (I.M.); christof.lenz@med.uni-goettingen.de (C.L.); henning.urlaub@med.uni-goettingen.de (H.U.); 3 Institute for Animal Breeding and Genetics, University of Veterinary Medicine Hannover, 30559 Hannover, Germany; Klaus.Jung@tiho-hannover.de; 4 Clinic for Gastroenterology and Gastrointestinal Oncology, University Medical Center Göttingen, 37075 Göttingen, Germany; draddat@gwdg.de; 5 Proteomics Group, Max-Planck-Institute of Experimental Medicine, 37075 Göttingen, Germany; jahn@em.mpg.de; 6 Bioanalytical Mass Spectrometry, Max Planck Institute for Biophysical Chemistry, 37075 Göttingen, Germany; 7 Laboratory of Biotechnology and Molecular Bioengineering, Faculty of Sciences and Techniques Gueliz, Cadi Ayyad University, 40000 Marrakech, Morocco; dihazi_abdel@yahoo.fr (A.D.); aelmeziane@gmail.com (A.E.M.); 8 Center for Biostructural Imaging of Neurodegeneration (BIN), University Medical Center Göttingen, 37075 Göttingen, Germany

**Keywords:** Diabetic nephropathy, early diagnosis/prognosis, E-cadherin, early biomarker

## Abstract

Diabetic nephropathy (DN) is the main reason for end-stage renal disease. Microalbuminuria as the non-invasive available diagnosis marker lacks specificity and gives high false positive rates. To identify and validate biomarkers for DN, we used in the present study urine samples from four patient groups: diabetes without nephropathy, diabetes with microalbuminuria, diabetes with macroalbuminuria and proteinuria without diabetes. For the longitudinal validation, we recruited 563 diabetic patients and collected 1363 urine samples with the clinical data during a follow-up of 6 years. Comparative urinary proteomics identified four proteins Apolipoprotein A-I (APOA1), Beta-2-microglobulin (B2M), E-cadherin (CDH1) and Lithostathine-1-alpha (REG1A), which differentiated with high statistical strength (*p* < 0.05) between DN patients and the other groups. Label-free mass spectrometric quantification of the candidates confirmed the discriminatory value of E-cadherin and Lithostathine-1-alpha (*p* < 0.05). Immunological validation highlighted E-cadherin as the only marker able to differentiate significantly between the different DN stages with an area under the curve (AUC) of 0.85 (95%-CI: [0.72, 0.97]). The analysis of the samples from the longitudinal study confirmed the prognostic value of E-cadherin, the critical increase in urinary E-cadherin level was measured 20 ± 12.5 months before the onset of microalbuminuria and correlated significantly (*p* < 0.05) with the glomerular filtration rate measured by estimated glomerular filtration rate (eGFR).

## 1. Introduction

Diabetic nephropathy (DN) is a common complication of diabetes and the leading cause of chronic kidney disease. In 2015, it was estimated that more than 415 million people worldwide are affected by diabetes mellitus. By 2040, the number will increase to 642 million [1], of which 90% will suffer from type 2 diabetes. Approximately 40% of patients with diabetes develop DN characterized by an increase in albuminuria and blood pressure, and a declining kidney function progressing to end stage renal disease [2]. Even mild degrees of albuminuria and a decrease in the glomerular filtration rate are associated with significantly increased risks of developing cardiovascular disease, end-stage renal disease, and/or premature mortality [3,4,5]. Microalbuminuria (MA) (urinary albumin excretion 30–299 mg/24 h or 30–299 mg albumin/g creatinine) is an independent predictor of overt nephropathy, loss of renal function and incidence of cardiovascular disease in patients with diabetes 2. Considering the increased incidence of diabetes and the resulting increased incidence of DN and the disadvantages of MA in revealing early tissue changes in the kidney, the detection of early signs of renal damage in diabetic patients is of paramount importance to provide timely therapy that slows down or even prevents evolution towards end-stage renal disease (ESRD). Early intervention in high-risk diabetes patients for the development of nephropathy is an attractive option, and a new meta-analysis shows the benefit of blocking of the renin-angiotensin-aldosterone system even in normo-albuminuric type 2 diabetic patients [6]. Despite this tremendous progress, the high intra-individual variability of urinary albuminuria and the lack of sensitive tests that can detect diabetic nephropathy prior to the onset of microalbuminuria still hinder early intervention [7,8,9,10,11]. Several treatable risk factors, such as arterial hypertension, poorly regulated diabetes mellitus, smoking, or increased excretion of urinary albumin within the normal range have been identified but are not sufficient to identify all those who will progress to DN [12]. Urinary proteomic studies have attracted significant attention because of their ability to identify small proteins and peptides associated with pathophysiological changes, particularly at an early stage of the disease [13,14] and because of the tremendous advances achieved in the recent years in the development of the mass spectrometric instrumentation and workflow. Several groups, including ours, conducted different studies to identify specific biomarker or biomarker patterns for DN. The tentatives resulted in a number of potential biomarkers [13,14,15]. Jiang and colleagues used a proteomic approach to investigate urinary proteome from diabetic patients with and without nephropathy and to compare them to healthy controls. Using small patient cohorts, they identified E-cadherin as discriminating protein between the diabetic groups and healthy controls [16]. Despite the large number of identified biomarkers in the previous studies, no one of them has made the way to bed side or into clinical trials. Moreover, the validation in larger patient cohort and in longitudinal studies is still missing and the advantage of these biomarkers compared to routinely used ones e.g., albuminuria and estimated glomerular filtration rate (eGFR) are still not sufficiently proven. Therefore, there is still a driving need for more robust, sensitive and specific biomarkers for earlier detection of diabetic patients at risk to develop renal damage and their longitudinal validation in a large number of patients. In the present study, we identified in a discovery phase four potential biomarkers for diabetic nephropathy, we validated the identified biomarkers with independent methods, and we proved the prognostic value of one of the makers in a longitudinal study. Moreover, we suggest a concentration threshold of this marker as risk predictor for nephropathy in diabetic patients.

## 2. Materials and Methods

### 2.1. Study Design

#### Patients

In the discovery study 47 patients were included and, for the validation phase of the study, 212 patients and 15 healthy individuals were recruited at the center of internal medicine of the University Medical Center Göttingen, Germany. Four patient groups were rigidly defined based on clinical course and urine parameters (proteinuria, albuminuria). For proteomic analysis, a healthy individual group (Ctr) was added to increase the specificity of potential urinary markers for DN. The patient groups for the validation included individuals with type II DM with microalbuminuria (DN Micro; *n* = 60), DM with macroalbuminuria (DN Macro; *n* = 60), patients with DM without micro- or macroalbuminuria (DM; *n* = 60), patients with proteinuria nondiabetic disease (NP; *n* = 32) (Table 1).

To validate the prognostic/predictive value of the biomarkers, 563 patients were recruited in a longitudinal study and, during the study period, 1363 urine samples were collected. The included patients met the following criteria: Type II diabetic patients without nephropathy (DM), type II diabetes mellitus patients diagnosed and treated according to national guidelines, eGFR > 60 mL/min, no microalbuminuria or macroalbuminuria [17]. Exclusion criteria were: active or chronic infectious disease, psychiatric comorbidity, pregnancy, drug- or pill-abuse, lack of consent, participation in another study, severe congestive heart failure (NYHA III-IV), active neoplastic comorbidity, pre-existing chronic kidney disease or circumstances rendering determination of the outcome difficult or impossible. The primary endpoint was the incidence of micro- or macroalbuminuria in DM patients. From this population we used for the longitudinal validation patients, who developed microalbuminuria during the study time (85 patients). Out of this group we selected patients with at least three follow-up urine samples, this resulted in 29 patients and 106 samples. Apart from clinical data (anamnesis, drugs, physical examination including blood pressure) additional laboratory parameters (HbA1c, serum creatinine, eGFR) were determined in recruiting centers.

### 2.2. Sample Collection and Handling

The clinical sample acquisition and analysis as well as the data management during this study were approved by the local Ethics Committee of the University Medical Center Göttingen, Germany (1/2/13). All patients had given their informed consent before the study. For all proteomics experiments, urine samples collection and treatments were performed according to our established protocol [14,18]. Midstream urine was collected in 15 mL tubes and centrifuged at 1000× *g* for 10 min at 4 °C to remove cell debris and casts. The supernatant was aliquoted into 2 mL aliquots and used immediately or stored at –80 °C until use. From each collected urine sample, we used 2 mL to measure routine laboratory parameters. All laboratory parameters were measured by standard routine methods in the certified University Medical Center Laboratories, Göttingen.

### 2.3. Depletion of High Abundant Proteins

Prior to protein depletion and two-dimensional gel electrophoresis (2-DE), sample enrichment was performed. For the discovery phase, urine samples from 47 patients (*n* = 10, DN Micro, *n* = 15, DN Macro, *n* = 10 DM, *n* = 12 NP) were used. Four different experimental groups were generated, with balanced number of samples in each group where possible. From each patient group, urine aliquots with equal protein amount (600 µg/aliquot) were pooled together and 10 mL of pooled urine were concentrated to 2 mL with a Vivaspin 20 Ultrafiltration Unit (Sartorius Göttingen, Germany). Sample aliquots with 1.6 mg urine proteins were used for depletion of high abundant protein and protein precipitation as described below.

Impaired glomerular filtration often leads to accumulation of high abundant serum proteins in the urine, as is the case in diabetic nephropathy. To enhance the detection of low abundant proteins in urine, concentrated urine samples were subjected to a depletion step targeting six major serum proteins using immunoaffinity chromatography. For this purpose, the pooled urine samples (1.6 mg each) were buffered with 10 mM Tris-HCl, pH 7.4 and loaded on a Human-6 affinity depletion column (Agilent, Santa Clara, California, USA). The matrix in the column is covalently bound with antibodies (against albumin, IgG, IgA, transferrin, haptoglobin and antitrypsin). During the chromatographic run, the low abundant proteins, not interfering with the antibodies, were washed out first. By switching the buffer to 100 mM glycin-HCl, pH 2.5, the captured high abundant proteins were eluted. The column was re-equilibrated using neutralization buffer 100 mM Tris-HCl, pH 8, before the next sample was applied. The chromatography was performed with an HPLC-system from Shimadzu, Kyoto, Japan. To ensure for reproducibility of the protocol triplicate from the same urine sample were depleted and two-dimensional protein patterns were generated. The amount of the proteins and their profiles were highly identical between the replicates as revealed by the overlay of 2-DE patterns confirming the robustness of the protocol.

### 2.4. Protein Precipitation and Concentration Estimation

The sample fractions with the low abundant proteins were subjected to protein precipitation to reduce the volume and enrich the proteins. The precipitation was carried out by adding 3 volumes of ice-cold acetone containing 10% methanol and incubating overnight at –20 °C. Precipitated proteins were pelleted by centrifugation at 12,000× *g* for 45 min at 4 °C. The pellets were dried and resolved in labeling buffer (30 mM Tris-HCl pH 8.5, 9.5 M urea, 2% CHAPS). The protein concentration was determined according to the Bradford method using BSA as calibrator [19].

### 2.5. Two-Dimensional Difference In-Gel Electrophoresis (2D-DIGE)

For 2D-DIGE, urinary protein depletion and precipitation were performed as described above. The labeling reaction was performed according to the manufacturer’s protocol (GE Healthcare, Munich, Germany). To control for dye-specific protein labeling, every pair of protein samples from two independent protein preparations were processed in duplicate while swapping the dyes. Thereby four replicate gels were obtained. The gels were scanned at 50 µm resolution on a Fuji FLA5100 scanner (Fuji Photo, Kanagawa, Japan). Image acquisition was carried out in 16-bit TIFF format. Delta2D 3.4 software (Decodon, Greifswald, Germany) was used for spot matching and normalization based on the internal standard. To analyze the significance of protein regulation, a Student’s t test was performed, and statistical significance was assumed for *p*-values less than 0.05. For further processing of the protein of interest, the gels were post-stained with colloidal Coomassie blue overnight. The protein spots of interest were then processed for mass spectrometric analysis and identification.

### 2.6. In Gel Digestion and Protein Identification from 2D-DIGE

Manually excised gel plugs were subjected to an automated platform for the identification of gel-separated proteins as described earlier [20,21]. The mass spectrometric analysis was performed according to our standard protocol. Database searches in the Swiss-Prot and NCBI primary sequence database were performed using the MASCOT Software 2.2 (Matrix Science, London, United Kingdom). The minimum requirement for accepting a protein as identified was at least one peptide sequence match above the identity threshold in addition to at least 20% sequence coverage in the peptide mass fingerprint (PMF).

### 2.7. Filter Aided Sample Prep (FASP)

FASP digestion and mass spectrometric analysis were performed according to Wisniewski et al. [22]. Briefly, urine sample aliquots from both discovery groups and follow up group were used for FASP protocol. After protein precipitation, 40 µg proteins from each sample was recovered in 200 µL buffer containing 8 M urea in 0.1 M Tris/HCl pH 8.5. The sample was then added to the filter unit (Vivaspin 20 Ultrafiltration Unit Sartorius Germany) and centrifuged for 15 min at 14,000× *g*. After discarding the flow-through from the collection tube, the urine proteome on the filter unit was incubated with 100 µL 0.05 M iodoacetamide in urea buffer for 20 min. Thereafter, the samples were centrifuged for 10 min at 14,000 rpm and washed twice each for 15 min with the urea buffer. Prior to the digestion step, the urinary proteome on the filter was equilibrated twice by adding 100 µL 0.05 M NH4HCO3 and centrifuging for 10 min at 14,000 rpm. The tryptic digestion step was performed overnight at 37 °C by adding 40 µL trypsin solution (12.5 ng/µL in 0.05 M NH4HCO3) to the proteins on the filter unit. The resulting tryptic peptides were extracted by centrifuging in a first step the filter unit at 14,000 rpm for 10 min. In a second step, 40 µL 0.05 M NH4HCO3 were added to the filter unit and centrifuged for 10 min. The extracted peptide mixture was acidified by the addition of trifluoroacetic acid (TFA) and dried in a speed vacuum centrifuge before processing for MS/MS analysis and label-free quantification.

### 2.8. Mass Spectrometry Analysis of the Extracted Tryptic Peptides from FASP and Spectral Counts Quantification

FASP tryptic digests derived from discovery urine groups were analyzed on a hybrid quadrupole-orbitrap mass spectrometer (Q Exactive, Thermo Fisher Scientific, Dreieich, Germany) and the tandem mass spectra extracted. All MS/MS samples were analyzed using Mascot (v2.4.1, Matrix Science, London, UK). Mascot was set up to search the SwissProt_2018_05 database (selected for Homo sapiens) with trypsin as digestion enzyme. Data was searched with a fragment ion mass tolerance of 0.020 Da and a parent ion tolerance of 10.0 ppm. Carbamidomethylation of cysteine was specified as a fixed modification and oxidation of methionine as a variable modification. Scaffold (version Scaffold_4.4.5, Proteome Software Inc., Portland, OR, USA) was used to validate MS/MS based peptide and protein identifications. After normalization, mass spectrometric samples were semi-quantitatively compared based on Total Spectral Counts (TSC) per protein. Peptide identifications were accepted if they could be detected with at probability greater than 80.0% to achieve a False Discovery Rates (FDR) of less than 1.0% using the Scaffold Local FDR algorithm. Protein identifications were accepted if an FDR of less than 1.0% could be achieved with a probability more than 28.0% and contained at least two identified peptides. Protein probabilities were assigned by the Protein Prophet algorithm. Proteins containing similar peptides and could not be distinguished by MS/MS analysis alone were grouped together to comply with the principles of parsimony [23]. Proteins sharing significant peptide evidence were grouped into clusters.

### 2.9. Mass Spectrometry and Label-Free Quantification of the Extracted Tryptic Peptides from FASP

For the urine samples from the follow-up study a label-free quantification of the FASP derived tryptic digest was performed as described in detail in supplemental material part.

### 2.10. Western Blot Analysis

For the validation of the proteomic data, the Western blot analyses according to Towbin et al. [24] were carried out. To compensate for sample pool error used for 2D-DIGE, Western blot analyses were performed from a single urine sample. From each experimental group (DM, DN Micro, DN Macro and NP), 24 individual urine samples per group were included in these analyses. Mouse monoclonal anti-REG1A (Abcam, U.K.), Rabbit monoclonal anti-CDH1 (Cell Signaling), mouse monoclonal anti-APOA1 (Abcam, U.K.) and mouse monoclonal anti-B2M antibodies (Abcam, U.K.), were used as primary antibodies. Molecular Probes Alexa Fluor 647 goat anti-mouse IgG antibody or Alexa Fluor 647 goat anti-rabbit IgG were used as secondary antibodies. The scanning of the blot membranes was carried out at 50 μm resolution on a Fuji FLA5100 scanner.

### 2.11. Dot Blot Analysis

To investigate the diagnostic value of identified proteins with an independent immunological method, we performed a dot blot analysis [18]. For this validation, individual samples from the large cohort (DM, DN Micro, DN Macro) 60 sample each group and 32 for NP group were used. Then, 10 µg of each urine sample was loaded in triplicate. Digitized dot blot images were further analyzed with Image J software (NIH, http://rsbweb.nih.gov/ij/).

### 2.12. ELISA-Analysis to Validate the Prognosis Value of the Biomarker

To validate the prognostic value of the CDH1 and APOA1 in longitudinal study, the urine CDH1 and APOA1 were measured by Human E-cadherin Quantikine or Human Apolipoprotein A-I Quantikine ELISA Kits respectively (R&D Systems, Minnesota USA). For this purpose, a subset of diabetic patients (who developed microalbuminuria during the study time and with at least three follow-up urine samples: 29 patients and 106 samples) was included from the longitudinal study group. After a collection time of 72 months, the samples were analyzed retrospectively to estimate the excretion level of CDH1 and APOA1 in urine. The used ELISA-kits have sensitivity values of 0.09 and 0.60 ng/mL respectively. Urine samples were diluted 20-fold in Calibrator Diluent RD5P. Urine concentrations were corrected for dilution using urine protein values.

### 2.13. Protein Immunoprecipitation and MALDI-TOF Mass Spectrometry Analysis

Immunoprecipitation of CDH1 from urine samples was performed using the protein G-agarose matrix and the anti-CHD1 antibody (Cell Signaling) according to our recent paper [25]. The measurement of the molecular weight of the immunoprecipitated proteins was carried out with a Rapiflex-MALDI-TOF-TOF mass spectrometer (Bruker, Bremen, Germany) on linear positive mode using sinapinic acid as matrix.

### 2.14. Statistical Methods

All statistical analyses were performed using the free software R (version 2.12, www.r-project.org). Metric parameters were compared between the study groups using either one-way analysis of variance (normal data) or the Kruskal-Wallis test (non-normal data). The normality was checked by quantile-quantile plots. Significant parameters were further investigated using either pairwise t-tests (normal data) or pairwise Mann-Whitney-U tests (non-normal data). The categorial parameters were compared using Fisher’s exact test between the study groups. Global tests were performed at a significance level of α = 0.05. Pairwise comparisons were performed at a Bonferroni-adjusted significance level of α* = 0.05/6 = 0.0083.

The diagnostic power of parameters that reached significance within pairwise comparisons was examined by receiver/operator characteristic (ROC) curves. ROC curves display sensitivity versus 1-specificity for each possible cutoff. Optimal cutoffs were determined according the Youden-criterion, i.e., by maximizing “sensitivity and specificity”. As measure for the diagnostic accuracy, the area under each ROC curve (AUC) was employed.

Dot blot and Western blot parameters, were combined by logistic regression models to improve classification accuracy. The stepwise variable selection was applied to remove correlated parameters from each model. The diagnostic power of these models was examined by leave-one-out cross validation. Optimal cutoffs for these models were again determined by ROC curve analyses.

## 3. Results

### 3.1. Clinical Parameters and Medications

Within the discovery and validation study population only systolic BP differed significantly between the three diabetic groups (*p* < 0.01), where patients of the DN Macro group had larger values than the DN Micro and the DM group. All other clinical parameters as well as the medications did not differ between the study groups (Supplementary Table S1).

In the subpopulation with Western blot results, neither the clinical parameters nor the medications differed significantly between the four groups (Supplementary Table S2).

### 3.2. Effect of Depletion of High Abundant Proteins

The high abundant proteins e.g., albumin in urine is considered as a drawback to the resolution of the 2D-DIGE. To overcome this obstacle, we used the Human-6 multiple affinity column and removed the six most abundant proteins from the urine. The procedure was carried out for all samples dedicated to 2D-DIGE. The comparative 2D electrophoresis (2-DE) analysis before and after depletion revealed a significant improvement in the access to low abundant proteins in urine (Supplementary Figure S1A). Moreover, replicate depletion of the same samples followed by 2-DE, showed high reproducibility of the protein pattern and confirm the robustness of the protocol.

### 3.3. Mapping DM-NP Urine Proteome and Identification of Potential Proteins Markers

**Figure 1:**
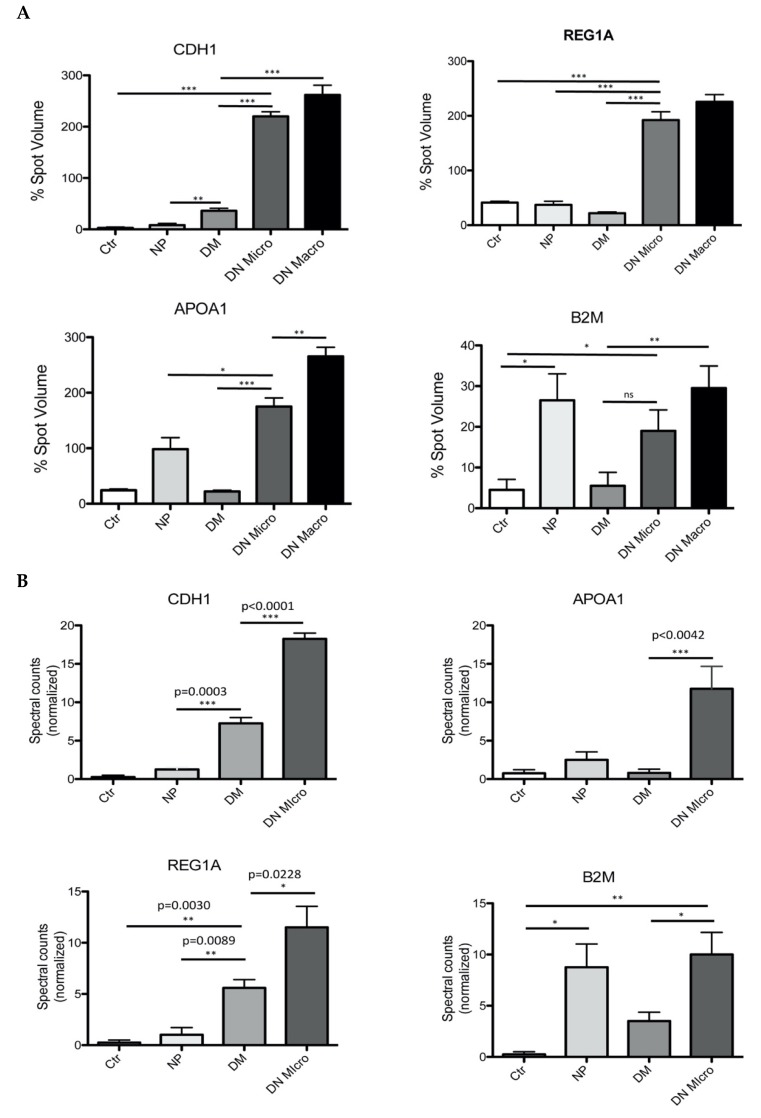
Two-Dimensional Difference In-Gel Electrophoresis (2D-DIGE) separation and quantification of the urine protein excretion level. (**A**) spot volume quantification of four proteins found (CDH1, REG1A, APOA1, B2M) to differentiate between the investigated groups. The protein excretion quantification for the four proteins is given in form of bar diagrams. Results are given as the means ± SD of the percentage volume of spot as quantified by 2D-DIGE. All the proteins showed significant changes in their excretion level between the four investigated groups (*p* < 0.05) (**B**) Mass spectrometric analysis and spectral account-based quantification of the protein of interest in urine of the four investigated groups. The software we used to quantify the protein abundance based on mass spectrometry is “Scaffold” (version Scaffold_4.4.5, Proteome Software Inc., Portland, OR, USA). MS/MS data are normalized between the samples; this allows a comparative analysis of the abundances of a protein between analyzed groups. All the proteins investigated, showed a significant level changes between the groups (*p* < 0.05). Ctr: healthy controls, NP: proteinuria without diabetes, DM: diabetic without nephropathy, DN Micro: diabetes with microalbuminuria, DN Macro: diabetes with macroalbuminuria.

For the 2D-DIGE analysis, samples from each group (*n* = 10, DN Micro; *n* = 15, DN Macro; *n* = 10, DM; *n* = 12, NP; Ctr., *n* = 15) were included to generate urine sample pools. To compensate for the disadvantage of urine pooling, three pools of urine from each group were generated. After protein depletion and precipitation, 2-DE were performed from each of the three pool from the same group. The resulting 2-DE patterns within the same group were compared with each other according to our former published protocol [18]. A comparative analysis of the 2-DE patterns did not show any significant differences between the pattern from the pools of the same group (data not shown) confirming the usefulness of the sample pooling for the 2-DE. To investigate differences in protein excretion and to identify potential biomarker of renal injury in patients with DN, 2D-DIGE maps were generated from each of the investigated groups. Delta2D analysis and quantification of the protein spots identify differences in protein excretion behavior between the investigated groups. A subset of four proteins discriminating significantly the DN Micro and DN Macro from the other groups attracted our attention (Figure 1A, Supplementary Figure S1B). The four proteins were identified as E-cadherin (CDH1) (DN Micro/NP *p* < 0.001, DN Micro/DM *p* < 0.001), Apolipoprotein A1 (APOA1) (DN Micro/NP *p* < 0.001, DN Micro/DM *p* < 0.001), Lithostathine-1-alpha (REG1A) (DN Micro/NP *p* < 0.001, DN Micro/DM *p* < 0.001) and Beta-2-microglobulin (B2M) (DN Micro/NP *p* < 0.05, DN Macro/DM *p* < 0.01) (Supplementary Table S3, Figure 1A).

### 3.4. Filter-Aided Sample Preparation (FASP) of the Urinary Proteome and MS-Based Analysis of the Potential Marker Identified by 2D-DIGE

The 2D-DIGE analysis revealed four interesting proteins, which could discriminate between the investigated groups, especially between the DN and the other groups. To support the 2D-DIGE data and to increase the confidence of the identified markers, FASP of urine proteome digestion and mass spectrometry analysis were performed as described above. The independent validation of the 2D-DIGE data confirmed the discriminating value of the identified proteins. CDH1 (DN Micro vs. DM *p* < 0.0001, DN Micro vs. NP *p* < 0.0003), as well as REG1A (DN Micro vs. DM *p* < 0.05 DN Micro vs. NP *p* < 0.01), could be confirmed as differentiation markers between DN Micro and other groups (Figure 1B). For B2M, significant differences in excretion levels could be confirmed for DN Micro vs. DM *p* < 0.05 and DN Micro vs. Ctr *p* < 0.01. B2M was also highly excreted in the proteinuria group without diabetes (Ctr vs. NP *p* < 0.05) (Figure 1B). For the four proteins of interest, the FASP-MS confirmed the data from 2D-DIGE and suggested the four proteins as potential markers for further validation.

### 3.5. Immunological Validation of the Four Identified Protein Markers

**Figure 2:**
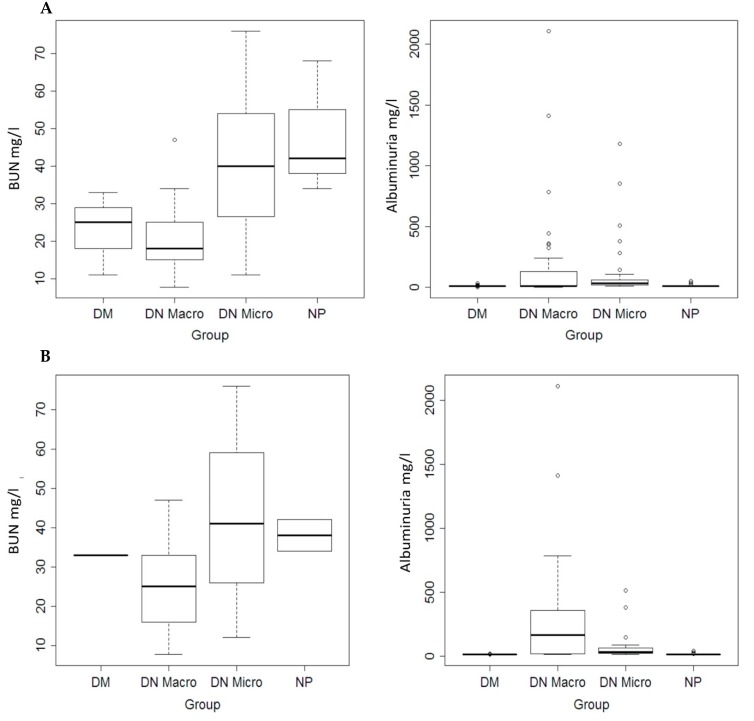
(**A**) Quantification of the blood urea nitrogen (BUN) values in complete study population (Left). Quantification of albuminuria in complete study population (right). (**B**) Quantification of the BUN (Left), and albuminuria (right) in Western blot patient subgroups.

Within the complete validation population, the albuminuria differed significantly (*p* < 0.01) between the study groups (Figure 2A, Table 1 and Table 2). The same was observed when only Western blot population subgroups were considered (Figure 2B, Table 1). Concerning the blood urea nitrogen (BUN) significant differences were observed only in dot blot population (Figure 2A,B, Table 1 and Table 2).

#### 3.5.1. Western Blot Validation

**Figure 3:**
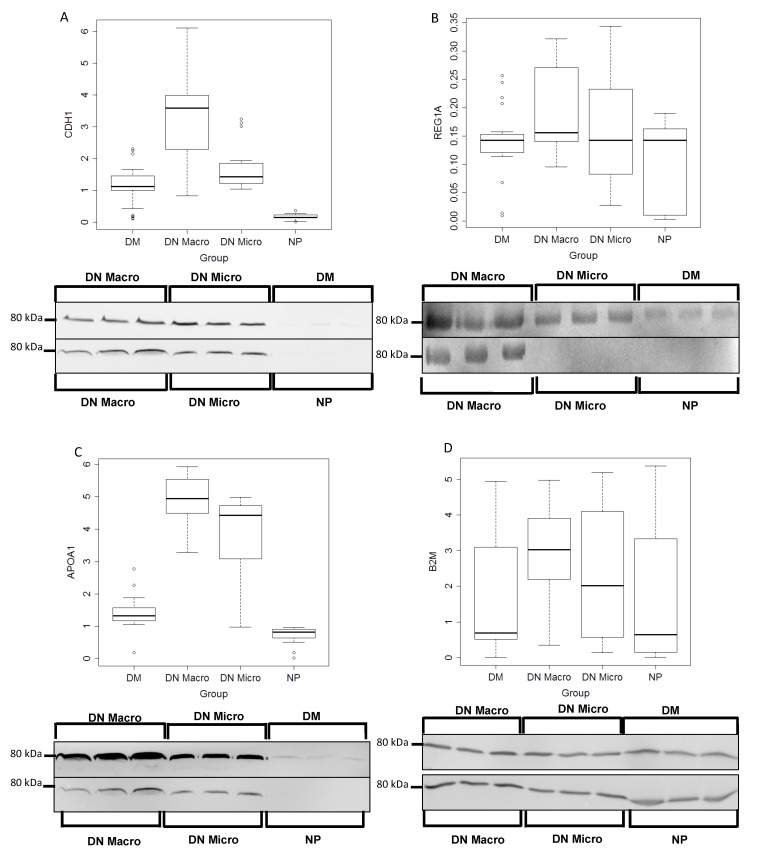
Western blot Analyses and quantification of the protein excretion in urine of potential marker. Urine samples from 24 patients each group (DM, DN Macro, DN Micro and NP) were collected and proteins were prepared as described in material and methods. CDH1, REG1A, APOA1 and B2M excretion levels in urine were quantified using fluorescence Western blot. Western blot quantification: on the y-axis the line-volume-percentage is given and the x-axis shows distribution of the intensity thought the corresponding urine group where the proteins were analyzed. (**A**) CDH1, (**B**) REG1A, (**C**) APOA1 and (**D**) B2M

To validate the discriminating value of the proteomics-identified markers, excretion levels of the four proteins in the various groups were examined using Western blot analyses. Urine samples *n* = 24 per group were used for this purpose, the excretion levels of the three proteins (CDH1, REG1A, APOA1) could differentiate significantly between the investigated groups (*p* < 0.05) (Figure 3A–D, Table 1). Whereas, the B2M could not be confirmed as discriminating marker.

#### 3.5.2. Dot Blot Validation of the Identified Markers

**Figure 4:**
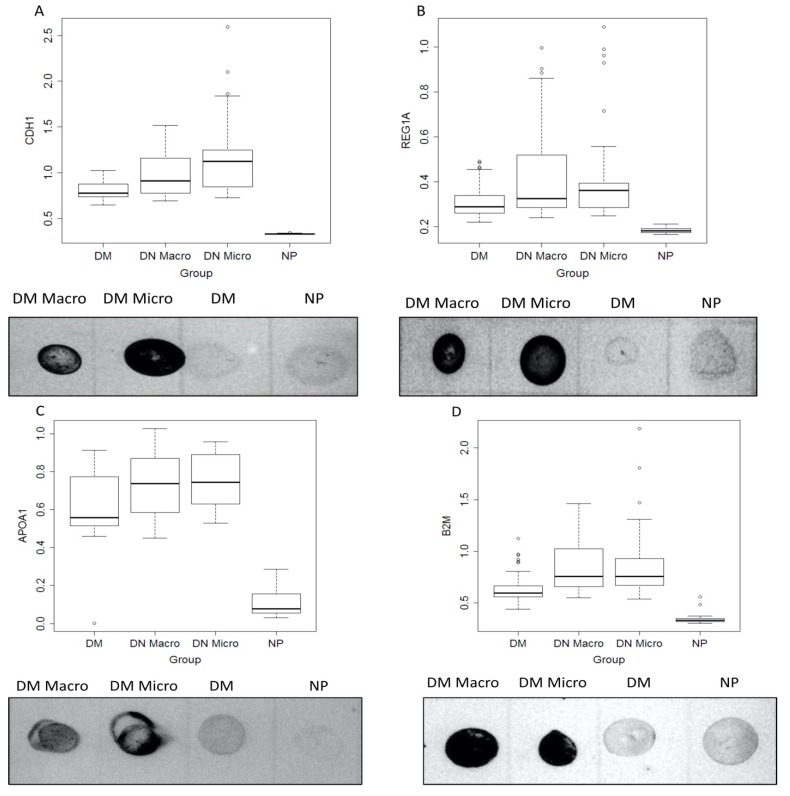
Dot blot analysis of the identified biomarker in larger patient’s cohort. (**A**,**B**) CDH1 and REG1A, (**C**,**D**) APOA1 and B2M. Urine samples from 60 patients each group (DM, DN Macro, DN Micro) and 32 for NP group were collected and proteins were prepared as described in material and methods. For dot blot analysis 10 µg from each sample were loaded in triplicate. On the y-axis the line-volume-percentage is given, and the x-axis shows investigated patients groups.

To confirm the usefulness of the identified markers, we validated the data with an independent immunological method. The extent of the protein excretion in the urine was tested by dot blot analysis in a large patient cohort. The dot blot quantification for APOA1, B2M, CDH1 and REG1A showed a significantly different distribution between the study groups (all *p* < 0.05) (Table 2). Statistical analysis of the dot blot data confirmed the increased excretion of the four proteins (CDH1, REG1A, APOA1, B2M) in urine samples from DN Micro and DN Macro compared to two other patient groups (*p* < 0.05) (Figure 4A–D, Table 2). Similar results were obtained in the samples analyzed by Western blot (Table 1). While BUN was no longer significant (*p* = 0.43), albuminuria and Western blot parameters were again significant (*p* < 0.01 for albuminuria APOA1, CDG1 and REG1A, and *p* = 0.02 for B2M). Detailed results of the subsequent pairwise comparisons are given in Supplementary Tables S4 and S5.

#### 3.5.3. Individual Diagnostic Power

**Figure 5:**
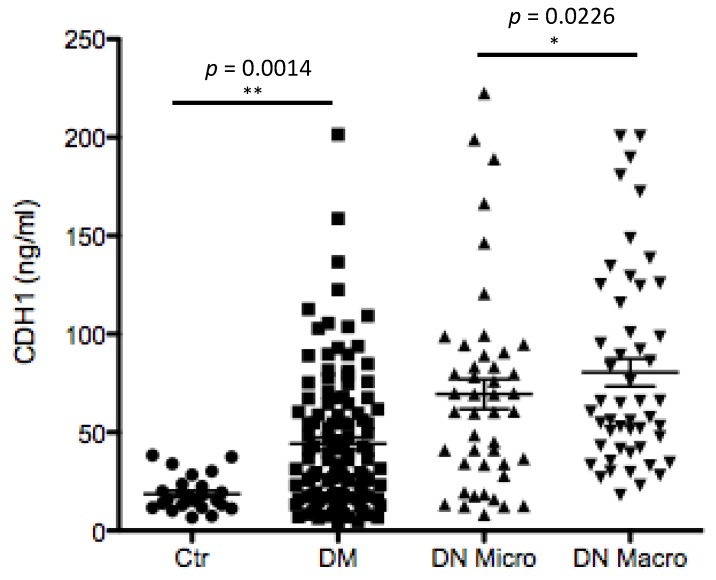
Enzyme-linked Immunosorbent Assay (ELISA) analyses and quantification of CDH1 in larger cohort of patients from four different groups. Urine samples from Ctr, DM, DN Micro and DN Macro were tested for the excretion level of CDH1 using ELISA. On the y-axis the measured protein concentration in ng/mL protein is given and the x-axis shows patients group. Statistical analyses were performed by Prizma4 software. The ELISA analysis showed a clear and highly significant difference in CDH1 excretion level between Ctr. and DM (*p* < 0.01) and between DM and DN Micro (*p* < 0.05).

In both the complete study population and in the Western blot subpopulation, some markers were able to separate completely the NP group from all other groups (Supplementary Table S6). For example, APOA1 (Dot blot result) yielded an AUC of 1.00 (95%-confidence interval: [1.00, 1.00]) in the ROC curve analysis of the NP versus DN Macro group. REG1A (Dot blot result) yielded the same AUC for the comparison of NP versus DM and of NP versus DN Micro. However, it was more difficult to differentiate DM patients from DN Micro or DN Macro patients with B2M levels (Dot blot). In the ROC curve analysis, an AUC of 0.79 (95%-CI: [0.75, 0.89]) was obtained comparing DM to DN Macro. An AUC of 0.88 (95%-CI: [0.81, 0.94]) was obtained for albuminuria in the comparison of DM versus DN Micro in the entire study population. Within the Western blot subpopulation, APOA1 scored the highest AUCs in discriminating DM from DN Micro or DN Macro patients (Supplementary Table S7). An ideal biomarker for DN should not only distinguish between DM and DN, but also discriminate between DN Micro and DN Macro. The most promising parameter here was CDH1, which yielded an AUC of 0.85 (95%-CI: [0.72, 0.97]). ELISA data confirmed the potential of CDH1 as discriminating parameter between different stages of nephropathy in diabetic patients (Figure 5). Moreover, the data revealed that the CDH1 as a potential marker for renal disfunction by diabetic patients fulfills also the criteria for a prognostic marker. The excretion level of CDH1 increased towards nephropathy, it ranges from 18.6 ± 8.7 ng/mL by healthy controls and increased significantly (>45 ng/mL) for DM patients at risk to develop nephropathy and to 77.8 ± 55.2 ng/mL for DN Macro (Figure 5).

#### 3.5.4. Combination of Parameters

The combination of parameters was studied only for those which were available with a comfortable sample size (i.e., did not have too many missing values). Table 3 and Table 4 show the combined models for selected pairwise classification problems. A combined model can only be considered as significantly better than a single parameter if the confidence interval for the AUC and the accuracy do not overlap. In this regard, none of these models were significantly better for the respective classification than one of the individual parameters.

### 3.6. Longitudinal Study

**Figure 6:**
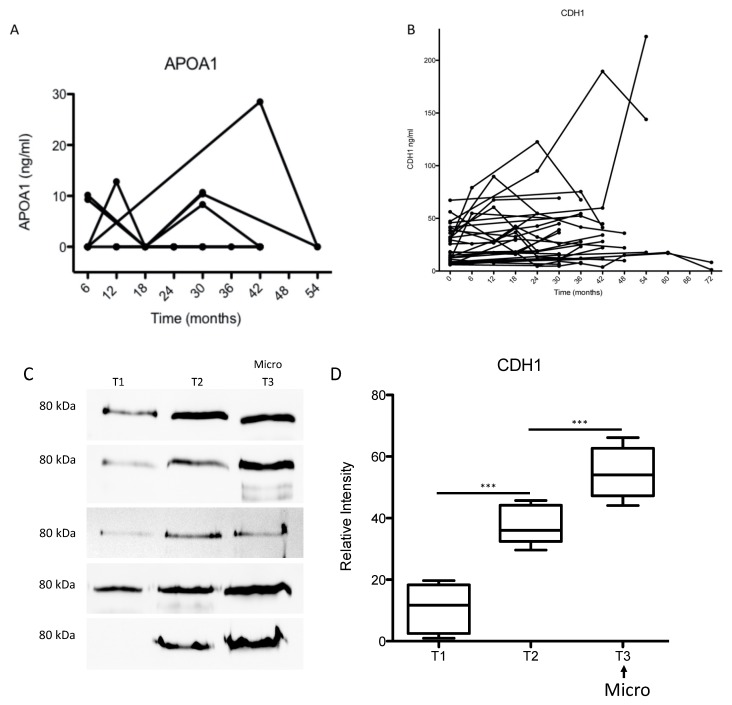
(**A**,**B**) ELISA analyses and quantification of CDH1 and APOA1 in flow-up samples. Urine samples from DM patients were collected over 72 months. The samples were retrospectively analyzed with ELISA to quantify CDH1 and APOA1 excretion level in urine. On the y-axis the measured protein concentration in ng/mL protein and the x-axis shows the urine collection time in months. Statistical analyses were performed by Prizma4 software. The quantification of the marker in the follow-up samples showed in large number of samples for CDH1 (**B**) a correlation between the excretion level and the progression towards microalbuminuria. Whereas for APOA1, the majority of the samples were negative for the proteins and the samples where APOA1 (**A**) was detected did not show any correlation between the amount of APOA1 and the progression towards nephropathy. (**C**,**D**) Western blot analyses and quantification of CDH1 in the follow-up cohort samples. Three follow-up urine samples were used for each patient. The three samples included the sample where the microalbuminuria was detected for the first time (T3) and two pre-microalbuminuric urine samples (T1 and T2). The collection times in this group was about 30 months, *** *p* < 0.001. T2 = T1 + 12–18 months; T3 = T2 + 6–12 months.

To validate the prognostic value of the four biomarkers, in particular CDH1, 563 patients were recruited in a longitudinal study and 1363 urine samples were collected during the study period (6 years). For the validation study, we used the patients who developed a microalbuminuria during the study period and had at least three follow up urine samples. This resulted in 29 patients and 106 samples. The age and gender distribution in the follow-up patient group is shown in the Supplementary Figure S2A,B. For three proteins, APOA1, REG1A and B2M, the excretion levels in the early stages of the disease were below the detection limit for ELISA in the majority of samples as exemplified for APOA1 (Figure 6A). In addition, label-free quantification confirmed the data from the ELISA and showed no significant correlation between the degree of excretion of these markers and the progression of albuminuria (Supplementary Figure S3B–D).

For the early prediction of DN and disease progression monitoring, CDH1 appears to be the most promising marker. To investigate whether CDH1 improves the early detection of DN compared to microalbuminuria and to test whether CDH1 can monitor the progression of kidney injury in diabetes, the excretion level of CDH1 was measured retrospectively in the urine samples of patients who developed microalbuminuria during the six years collection period in the longitudinal follow-up study. Compared to baseline, the data revealed an early increase in urinary CDH1 excretion in patients, at risk of developing microalbuminuria. The significant increase in urinary CDH1 levels was measured 20 ± 12.5 months before the onset of microalbuminuria. In the majority of patient samples (>70%) there was a positive correlation between the CDH1 urinary level and the progression toward microalbuminuria (Figure 6B–D). When modeling the renal function as described by eGFR as function of the urinary albumin excretion as well as CDH1 using a linear mixed model accounting for the clustered data structure within patient (Table 5), CDH1 is significantly related to the eGFR parameter (*p* < 0.05), whereas the urinary albumin shows no correlation with eGFR in early stages of nephropathy (Table 5, Supplementary Figure S3A).

## 4. Discussion

The increasing number of diabetic patients is directly reflected in the number of patients with DN with a prevalence of 20–40%. DN is the main reason for kidney failure and for the increasing number of patients receiving renal replacement therapy [26,27,28]. These result in enormous socio-economic problems and draw attention to the improvement of the management of this disease. Currently the management of DN consist of using inhibitor for renin-angiotensin-aldosterone system, beside the control of the hyperglycemia, blood pressure and dyslipidemia. These strategies are more beneficial if they are applied in early stages of the disease, and their use is less effective in an advanced stages of kidney injury. Alterations in albumin excretion are considered as the hallmark for prediction of the onset and progression of diabetic nephropathy [29,30,31,32]. Recent studies have demonstrated that deterioration in kidney function and pathological alteration in renal histology were also found in diabetes patients with normoalbuminuria. This indicates that albumin is not the perfect marker for DN [33]. Early detection of changes in renal function is of great benefit for the treatment of DN.

In our study, both proteomics methods, gel-based and gel-free assays, identified with high sensitivity and specificity E-cadherin encoded by CDH1 as a discriminating protein between DN and other groups. In addition, urinary CDH1 excretion levels were able to differentiate between the different stages of DN. Similar to our 2D-DIGE data, Jiang et al. used a gel-based proteomic approach to compare the urinary proteome of DN patients to the one of healthy volunteers, and identified with high statistical significance CDH1 as a discriminating factor between the two groups [16]. The expression of CDH1 was also investigated in DN patients’ tissues and was found to be downregulated compared to healthy control tissues [16]. In our study the Nephroseq (The Regents of The University of Michigan, Ann Arbor, MI) was used to analyze and visualize the CDH1 expression data in kidney tissue from two biopsy cohorts: small cohort with 22 samples and and large cohort with 201 biopsies belonging to different diseases groups (Supplementary Figure S4A,B). In the small cohort the data showed a significant up-regulation of CDH1 expression in the DN group compared to the other three groups (Supplementary Figure S4A), whereas in large group no significant regulation of CDH1 was observed (Supplementary Figure S4B) revealing that issue of the expression regulation of CDH1 in diabetic nephropathy still needs further investigations.

By quantifying the urine E-cad level in different groups in a large number of patients and controls, we were able to establish a critical urinary excretion threshold for urinary CDH1 as an indicator of renal function alteration. Our data suggest the following urinary excretion thresholds for CDH1: in healthy volunteers, the range of CDH1 excretion level is about 18.6 ± 8.7 ng/mL, whereas in diabetic patients at risk of developing nephropathy, the excretion level increases in early stage to >40 ng/mL. Interestingly, the majority of diabetic patients with an increased urinary CDH1 level developed within 36 months albuminuria. The label-free quantification and immunological data form the longitudinal follow-up study demonstrated a strong correlation between the urinary CDH1 levels and the progression toward nephropathy in diabetic patients. Moreover, the statistical analyses of immunological data form the longitudinal study demonstrate a strong correlation between urinary CDH1 levels and eGFR, which qualify this protein as a marker for the early detection of kidney function deterioration in diabetic patients.

**Figure 7:**
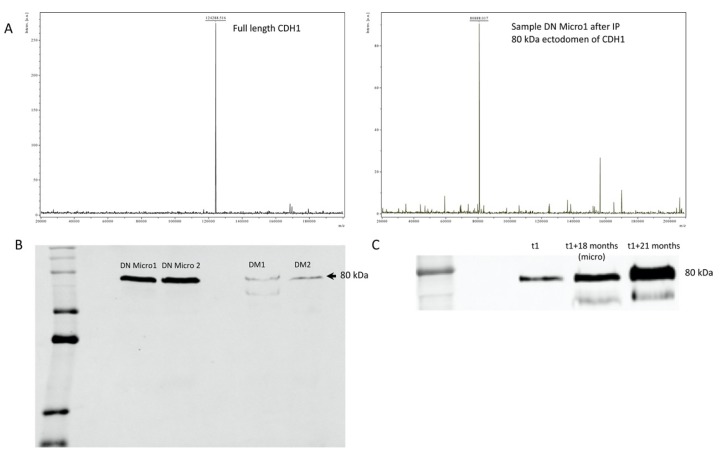
MALDI -TOF and Western blot investigation of the molecular weight of the urine soluble CDH1. (**A**) Urine samples, where the ELISA analysis revealed high level of CDH1 were used for immunoprecipitation and subsequent mass spectrometric analysis. The MALDI-TOF analysis was performed in linear mode with sinapinic acid as matrix. The mass spectra revealed that in contrast to the full length CDH1 (MW = 124 kDa), the urine soluble one has a lower molecular weight (MW = 80 k Da). (**B**) The Western Blot analysis confirmed the mass spectrometric data and showed a protein with MW = 80 kDa when tested with antibody against CDH1. (**C**) The Western blot analysis of samples from the flow-up study confirmed the increase in CDH1 urine level toward nephropathy and highlight the prognostic value of CDH1 for diabetic nephropathy.

E-cadherin (also called epithelial cadherin or uvomorulin) is a 120 kDa transmembrane glycoprotein, which plays an important role in the morphogenesis and wound healing and is involved in cell-cell adhesion in a calcium-dependent manner. In epithelial cells, E-cadherin forms a complex with catenin to stabilize intercellular adhesion and preserve the integrity of epithelial cells [34]. E-cadherin is coded by the CDH1/E-cadherin gene located in chromosome 16q22.1 [35,36]. E-cadherin is composed of an extracellular part with 5-extracellular cadherin domains linked to a cytoplasmic part [34], E-cadherin is not only involved in cell–cell adhesion, but also interacts with and modulates several signal transduction pathways [34]. Changes in E-cadherin expression or regulation of its function was described in a wide variety of diseases, particularly in carcinogenesis [37,38]. In this case, the lack of E-cadherin function results in an increase in cell proliferation, which favors the development of cancer [39]. It is also known that E-cadherin can be excreted in urine in soluble form [40]. The excreted form has been described by several groups as a marker of malignant progression, as in bladder cancer [41]. Moreover, soluble E-cadherin in serum serves as cancer prognostic marker [42] and the soluble fragment in urine corresponds to the 80 kDa ectodermal fragment of the protein, which can be cleaved by several proteases including plasmin, MMP3, MMP7, ADAM10, and ADAM-15 [43,44,45]. Cancer patients present high levels of soluble E-cadherin fragments in serum and urine, which was associated with a poor prognosis [40,42,46]. To investigate whether the urinary soluble E-cadherin in DN corresponds to an 80 kDa fragment, we combined immunoprecipitation with MALDI-TOF mass spectrometry. The mass spectra clearly showed an 80 kDa protein, which could be enriched from urine of DN patients with microalbuminuria (Figure 7A). In comparison, MALDI-TOF mass spectrometry analysis from commercial full-length E-cadherin displays a protein of 124 kDa corresponding to the molecular weight of the intact protein. Western blot analysis confirmed the data from mass spectrometry (Figure 7B) moreover they showed that in urine samples from the follow-up study, the amount of the 80 kDa E-cadherin fragment increased towards microalbuminuria, confirming the prognostic value of this protein (Figure 7C). The identified urinary E-cadherin in patients with DN corresponds to the 80 kDa ectodomain of E-cadherin, indicating a deterioration in the renal tubule due to diabetes.

With our study we confirmed that the CDH1 has the potential to serve as an early diagnostic/prognostic marker for diabetic patients at risk of developing kidney disease. Moreover, the release of CDH1 in the urine of DM patients seems to be an early indication of kidney function deterioration and epithelial damage.

## Figures and Tables

**Table 1:** Urinary and blood parameters compared between the study groups of the subpopulation with Western blot analysis.

Parameter	DM (*n* = 24)	DN Macro (*n* = 24)	DN Micro (*n* = 24)	NP (*n* = 24)	*p*
Creatinin (mg/dL)	0.83 ± 0.28	1.04 ± 0.39	1.03 ± 0.44	0.91 ± 0.32	0.18
BUN (mg/dL)	33.0 ± n.a.	25.3 ± 12.4	42.5 ± 26.2	38.0 ± 5.7	0.43
Albuminuria (mg/L)	11.7 ± 1.7	437.3 ± 682.3	73.1 ± 120.1	14.0 ± 6.8	<0.01
HbA1c (%)	7.4 ± 1.0	7.6 ± 1.2	8.2 ± 0.8	–	0.10
APOA1	1.4 ± 0.5	5.0 ± 0.6	3.8 ± 1.2	0.7 ± 0.2	<0.01
B2M	1.7 ± 1.6	3.0 ± 1.2	2.3 ± 2.0	1.6 ± 2.0	0.02
CDH1	1.1 ± 0.6	3.3 ± 1.4	1.6 ± 0.6	0.2 ± 0.1	<0.01
REG1A	0.14 ± 0.06	0.19 ± 0.07	0.16 ± 0.08	0.10 ± 0.07	<0.01

**Table 2:** Urinary and blood parameters, and the value of the biomarker (measured by Dot blot analysis) compared between the study groups.

Parameter	DM (*n* = 60)	DN Macro (*n* = 60)	DN Micro (*n* = 60)	NP (*n* = 32)	*p*
Creatinin (mg/dL)	0.92 ± 0.36	0.95 ± 0.33	1.07 ± 0.62	0.96 ± 0.42	0.39
BUN (mg/dL)	23.0 ± 11.1	20.3 ± 9.1	40.0 ±21.0	48.0 ± 17.8	< 0.01
Albuminuria (mg/L)	12.0 ± 4.5	195.1 ± 470.9	98.0 ± 210.9	14.7 ± 9.6	< 0.01
HbA1c (%)	7.4 ± 0.9	7.6 ± 1.2	8.2 ± 0.9	–	0.05
APOA1	0.62 ± 0.17	0.73 ± 0.16	0.76 ± 0.14	0.11 ± 0.07	<0.01
B2M	0.63 ± 0.14	0.85 ± 0.24	0.84 ± 0.28	0.34 ± 0.05	<0.01
CDH1	0.81 ± 0.10	0.97 ± 0.20	1.13 ± 0.34	0.33 ± 0.005	<0.01
REG1A	0.31 ± 0.07	0.43 ± 0.20	0.39 ± 0.18	0.18 ± 0.01	<0.01

**Table 3:** Combinations of Western blot parameters for selected pairwise classification problems. Shown cutoffs are designated for the logistic model e^M^/(1 + e^M^).

Classification: DM versus DN Micro Model: M = –13.44 + 3.22 * APOA1 + 0.87 * B2M + 27.25 * REG1A Cutoff: 0.30, Accuracy: 85 [72, 94], Sensitivity: 83 [63, 95], Specificity: 88 [68, 97]
Classification: DN Micro versus DN Macro Model: M = 27.57 –4.51 * APOA1 –0.47 * B2M –2.14 * CDH1 Cutoff: 0.20, Accuracy: 88 [75, 95], Sensitivity: 100 [86, 100], Specificity: 75 [53, 90]

**Table 4:** Combinations of Dot blot parameters for selected pairwise classification problems. Shown cutoffs are designated for the logistic model e^M^/(1 + e^M^).

Classification: DM versus DN Macro Model: M = –12.49 + 6.03 * APOA1 + 4.51 * B2M + 5.74 * CDH1 Cutoff: 0.50, Accuracy: 76 [68, 84], Sensitivity: 72 [59, 83], Specificity: 80 [67, 89]
Classification: DM versus DN Micro Model: M = –18.91 + 9.24 * APOA1 + 5.05 * B2M + 9.48 * CDH1 Cutoff: 0.58, Accuracy: 85 [77, 91], Sensitivity: 79 [66, 89], Specificity: 90 [79, 96]
Classification: DN Micro versus DN Macro Model: M = –1.99 + 2.801 * CDH1 -2.34 * REG1A Cutoff: 0.55, Accuracy: 69 [59, 77], Sensitivity: 53 [39, 66], Specificity: 84 [73, 93]

**Table 5:** In the analyzed samples from the diabetes follow-up cohort the E-cadherin correlated significantly with eGFR in early stages of nephropathy. Albumin did not show any significant correlation with eGFR in early stages of diabetic nephropathy.

	Estimate	Std. Error	df	t Value	Pr(>|t|)
(Intercept)	69.13	3.493	62.85	19.79	<0.0001
AlbU	0.01262	0.01383	76.49	0.9125	0.364
E-Cadherin ng/mL	0.04521	0.0179	77.46	2.525	0.013

## Supplementary Materials

The following are available online at https://www.mdpi.com/2077-0383/9/3/639/s1, Supplementary Figure S1: Two-dimensional pattern of total protein isolated from DN patient’s urine, Supplementary Figure S2, Supplementary Figure S3: Mass spectrometric analyses and quantification of the four-potential biomarker in the follow-up samples. Supplementary Figure S4, Supplementary Table S1: Clinical baseline parameters and medications compared between the study groups, Supplementary Table S2: Clinical baseline parameters and medications compared between the study groups of the subpopulation with Western blot analysis, Supplementary Table S3: Protein identification, the four differentially excreted proteins between the investigated groups, Supplementary Table S4: Pairwise group comparisons of significant parameters in the complete study population, Supplementary Table S5: Pairwise group comparisons of significant parameters in the subpopulation with Western blot analysis, Supplementary Table S6: Results of ROC curve analyses to distinguish between two of the study groups using different parameters, Supplementary Table S7: Results of ROC curve analyses to distinguish between two of the study groups using different parameters.
