# Supplementary material for: Urine E-cadherin: A Marker for Early Detection of Kidney Injury in Diabetic Patients
Source: J Clin Med. 2020 Feb 27;9(3):639. doi: 10.3390/jcm9030639 (PMC7141221; doi:10.3390/jcm9030639)
Supplement: Supplementary file 1 [file jcm-09-00639-s001.pdf]

MS sample preparation

For generation of a peptide library, equal amount aliquots from each sample were pooled to a total amount of 80 µg, and separated into eight fractions using a reversed phase spin column (Pierce High pH Reversed-Phase Peptide Fractionation Kit, Thermo Fisher Scientific). All samples were spiked with a synthetic peptide standard used for retention time alignment (iRT Standard, Schlieren, Schweiz).

Protein digests were analyzed on a nanoflow chromatography system (Eksigent nanoLC425) hyphenated to a hybrid triple quadrupole-TOF mass spectrometer (TripleTOF 5600+) equipped with a Nanospray III ion source (Ionspray Voltage 2400 V, Interface Heater Temperature 150 °C, Sheath Gas Setting 12) and controlled by Analyst TF 1.7.1 software build 1163 (all AB Sciex). In brief, peptides were dissolved in loading buffer (2% acetonitrile, 0.1% formic acid in water) to a concentration of 0.3 µg/µl. For each analysis, 1.5 µg of digested protein were enriched on a precolumn (0.18 mm ID x 20 mm, Symmetry C18, 5 µm, Waters, Milford/MA, U.S.A) and separated on an analytical RP-C18 column (0.075 mm ID x 250 mm, HSS T3, 1.8 µm, Waters) using a 55 min linear gradient of 5–35 % acetonitrile/0.1% formic acid (v:v) at 300 nl min-1.

Qualitative LC/MS/MS analysis was performed using a Top25 data-dependent acquisition method with an MS survey scan of m/z 350–1250 accumulated for 350 ms at a resolution of 30,000 full width at half maximum (FWHM). MS/MS scans of m/z 180–1600 were accumulated for 100 ms at a resolution of 17,500 FWHM and a precursor isolation width of 0.7 FWHM, resulting in a total cycle time of 2.9 s. Precursors above a threshold MS intensity of 125 cps with charge states 2+, 3+, and 4+ were selected for MS/MS, the dynamic exclusion time was set to 30 s. MS/MS activation was achieved by CID using nitrogen as a collision gas and the manufacturer's default rolling collision energy settings. Four technical replicates per reversed phase fraction were analyzed to construct a spectral library.

For quantitative SWATH analysis, MS/MS data were acquired using 65 variable size windows [1] across the 400-1,050 m/z range. Fragments were produced using rolling collision energy settings for charge state 2+, and fragments acquired over an m/z range of 350–1400 for 40 ms per segment. Including a 100 ms survey scan, this resulted in an overall cycle time of 2.75 s. Two replicate injections were acquired for each biological sample.

Protein identification was achieved using ProteinPilot Software version 5.0 build 4769 (AB Sciex) at "thorough" settings. A total of 230,975 MS/MS spectra from the combined qualitative analyses were searched against the UniProtKB human reference proteome (revision 02-2017, 92,928 entries) augmented with a set of 52 known common laboratory contaminants to identify 619 proteins at a False Discovery Rate (FDR) of 1%.

Spectral library generation and SWATH peak extraction were achieved in PeakView Software version 2.1 build 11041 (AB Sciex) using the SWATH quantitation microApp version 2.0 build 2003. Following retention time correction using the iRT standard, peak areas were extracted using information from the MS/MS library at an FDR of 1% [2]. The resulting peak areas were then summed to peptide and finally protein area values, which were used for further statistical analysis.

Statistical analysis was performed in Perseus Software v1.5.6.0 (Max Planck Institute for Biochemistry) [3]. Protein area values were log2-transformed and normalized by z-scoring. Protein abundance profiles were evaluated for similarity by Pearson correlation analysis.

Supplementary Figures

A

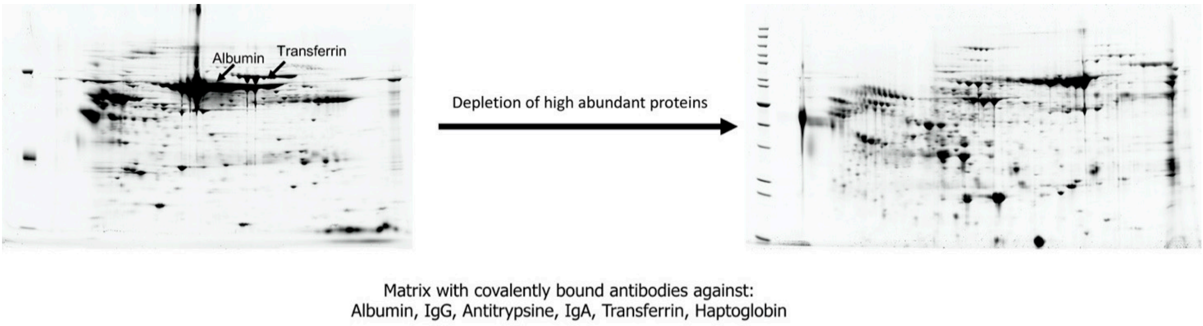

B

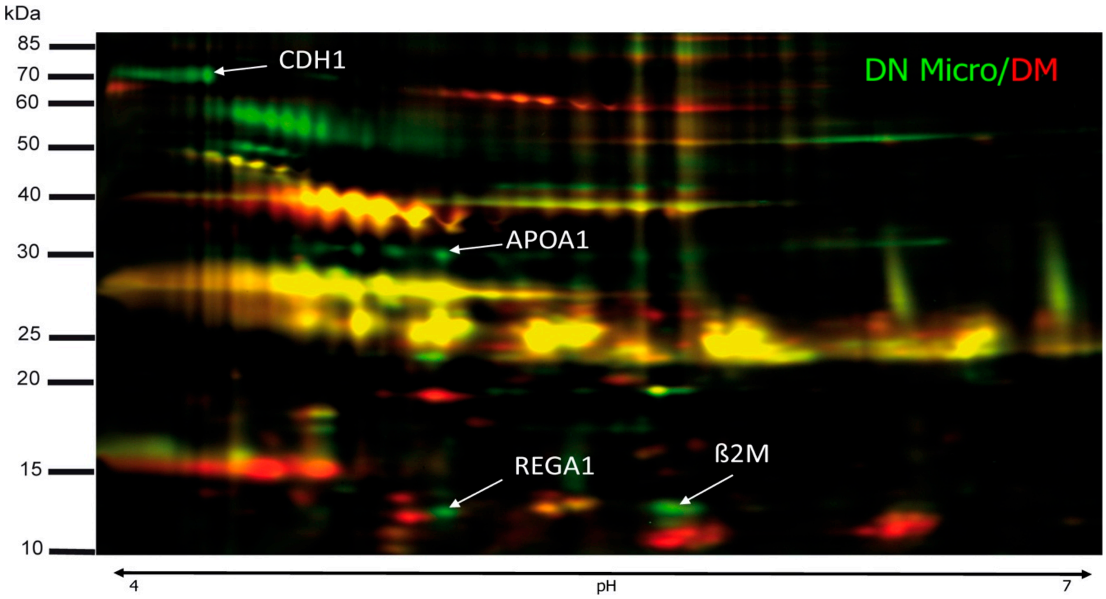

Supplemental Figure 1. Two-dimensional pattern of total protein isolated from DN patient`s urine. A: The proteins (150 µg) were loaded and separated by 2-DE according to pI and MW. A 11-cm IPG strip with a linear pH 4–7 gradient for isoelectric focusing, and a Criterion Tris-HCl Linear gradient gel 10-20 % for SDS-PAGE were used. The protein spots were visualized by Flamingo. Left: the protein pattern before depletion of the 6 high abundant proteins with Agilent Human 6 column. Right: the protein pattern of the same sample after depletion. The 2D gel pattern showed clearly the advantage of the depletion of high abundant proteins for getting access to low abundant ones. B: DIGE of urine protein samples from DN-Micro (green) and DM (red) after depletion of the high abundant proteins. The four proteins found to be differentially excreted are labeled with gene names on the gel. All the protein showed presents significant excretion level changes between DN Micro and DM (P<0.05)

A

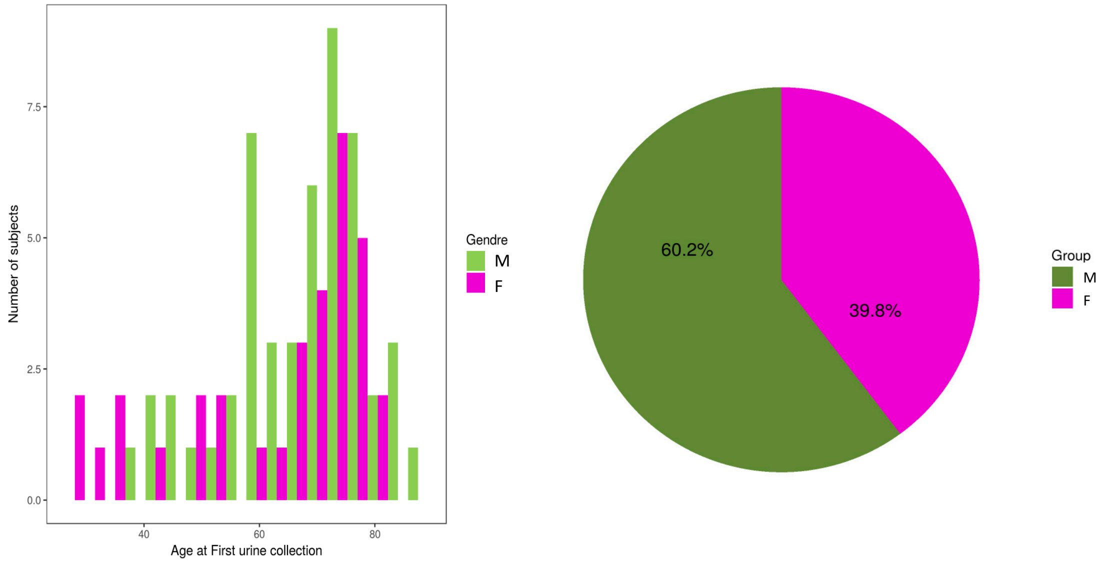

B

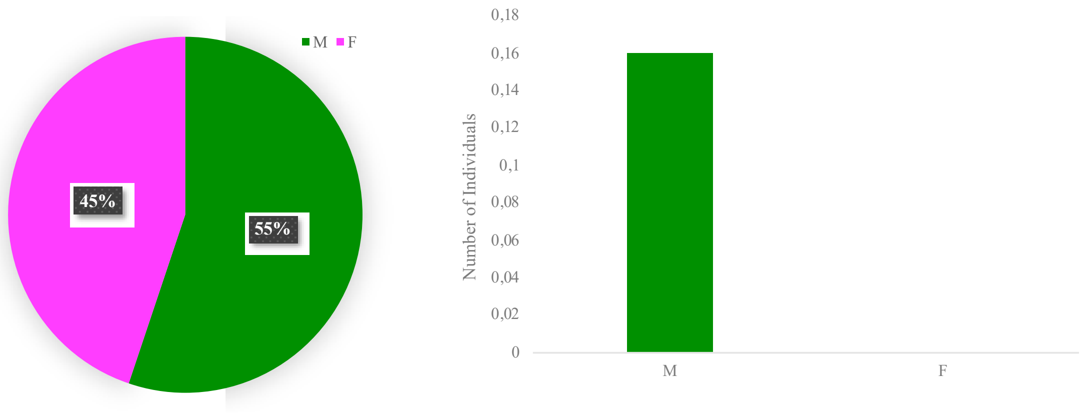

Supplemental Figure 2. A: Age and gender distribution of the patient's which developed microalbuminuria during the collection time. B: Gender distribution of the 29 selected patients (microalbuminuria and at least three urine samples during the collection time) for the ELISA validation in the follow-up study. M: male F: female

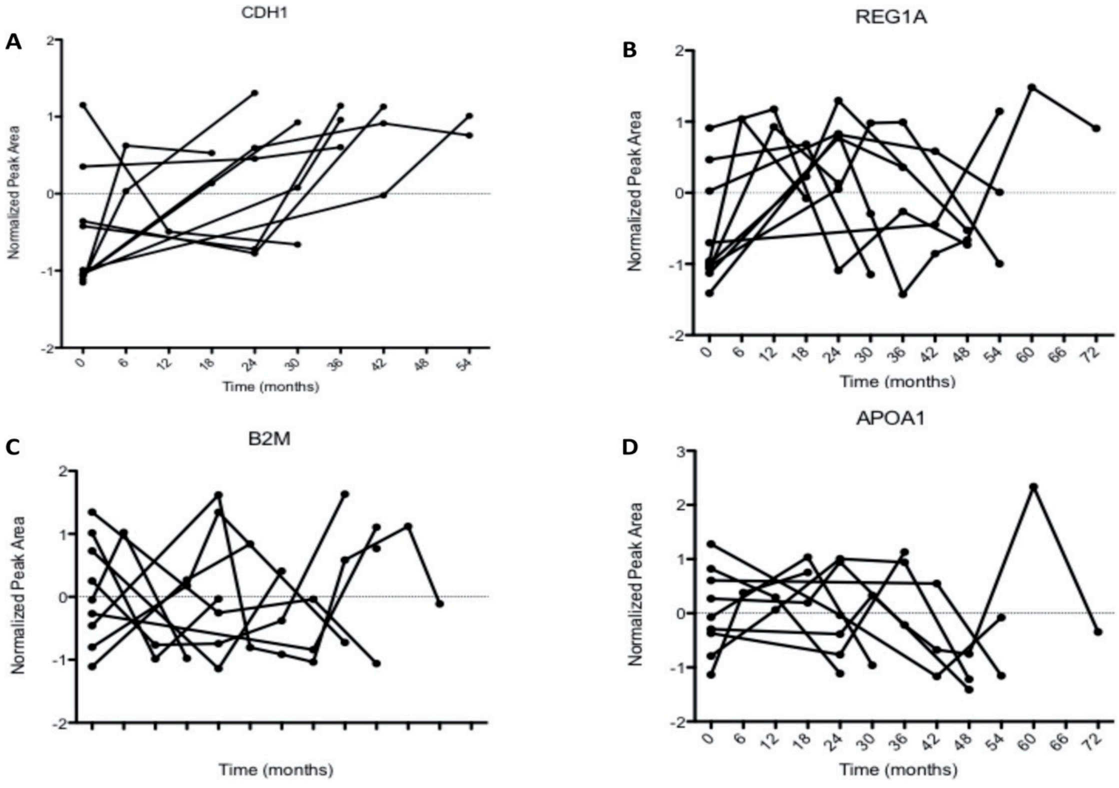

Supplemental Figure 3. Mass spectrometric analyses and quantification of the four-potential biomarker in the follow-up samples. Urine samples from DM patients were collected over 72 months. Parallel to the ELISA analysis, a mass spectrometry-based analysis and quantification was performed as described in material and methods part. The excretion level of the four proteins was quantified using the spectral account resulting from each biomarker. Statistical analyses were performed by Prizma4 software. The mass spectrometric quantification of the marker in the follow-up samples showed that the excretion level of CDH1 correlates with the progression toward nephropathy, whereas the excretion level of the other markers did not show any clear correlation. A: CDH1, B: REG1A, C: B2M, D: APOA1

A

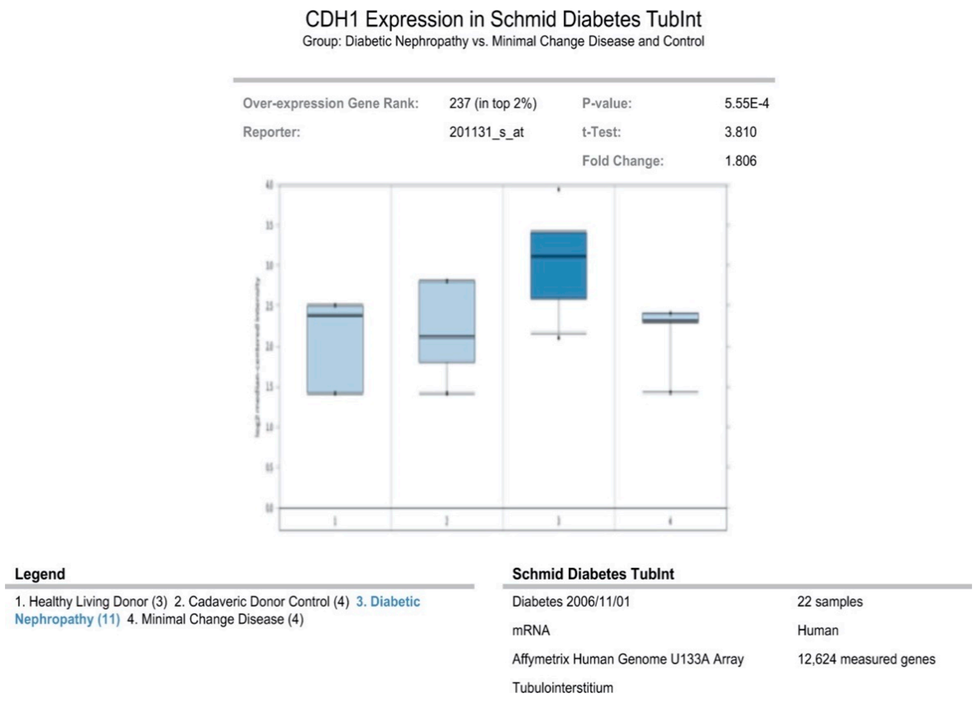

B

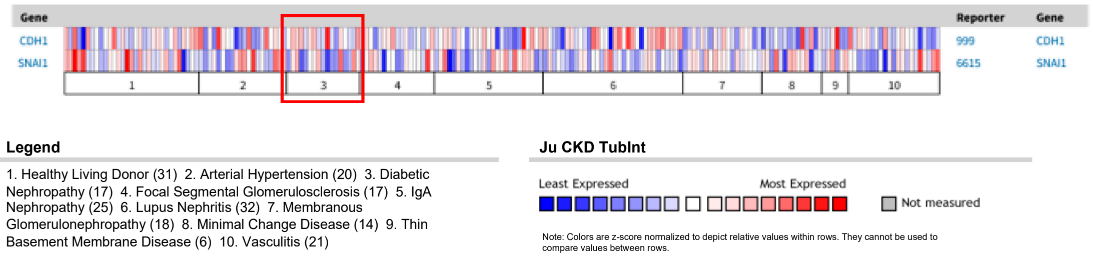

Supplemental Figure 4. A: Gene expression analysis of the identified biomarker in kidney tissue. Using the Nephroseq database for transcriptomics data, we investigated the expression of the CDH1 in kidney tissue of diabetic nephropathy patients and compared them to other groups [4]. B: Using Nephroseq database for we investigated the expression regulation of CDH1 and SNAIL1 in the transcriptomic data from Ju W. and colleagues [5].

Supplemental Tables

Supplemental Table 1 Clinical baseline parameters and medications compared between the study groups. *) p-value for diabetes type is only related to the first three groups.

**Parameter**	**DM** (n = 60)	**DN Macro** (n = 60)	**DN Micro** (n = 60)	**NP** (n = 32)	**p**
Age (years)	62.7 +/- 13.4	57.5 +/- 16.6	58.0 +/- 18.8	61.4 +/- 15.3	0.28
Sex					
Male	(25 41%)	28 (46%)	26 (43%)	15 (48%)	0.90
Female	(35 59%)	32 (54%)	34 (57%)	17 (52%)	
BMI	29.5 +/- 4.8	30.3 +/- 5.6	30.1 +/- 5.4	n.a.	0.88
Diabetes type					
1	(22 37%)	15 (25%)	25 (42%)	0 (0%)	0.55*
2	(38 63%)	45 (75%)	35 (58%)	0 (0%)	
none	0 (0%)	0 (0%)	0 (0%)	32 (100%)	
Arterial hypertension	16 (27%)	21 (35%)	18 (30%)	1 (3%)	0.64
Systolic BP (mmHg)	135.9 +/- 13.4	150.6 +/- 17.3	135.1 +/- 17.6	n.a.	< 0.01
Diastolic BP (mmHg)	76.2 +/- 10.7	79.9 +/- 13.1	76.6 +/- 12.3	n.a.	0.60
ACE inhibitors	(23 38%)	16 (26%)	12 (20%)	1 (3%)	0.58
AT1 inhibitors	5 (8%)	10 (17%)	8 (13%)	0 (0%)	0.79
Renin A	0 (0%)	1 (1.6%)	1 (1.6%)	0 (0%)	1.00
Ca Antagonist	6 (10%)	3 (5%)	9 (15%)	0 (0%)	0.59
Diuretics	6 (10%)	12 (20%)	13 (22%)	1 (3%)	0.30
Beta blocker	7 (12%)	14 (23%)	13 (21%)	1 (3%)	0.41
Antihypertensives	6 (10%)	10 (17%)	6 (10%)	n.a.	0.61

Supplemental Table 2 Clinical baseline parameters and medications compared between the study groups of the subpopulation with Western blot analysis). *) p-value for diabetes type is only related to the first three groups.

**Parameter**	**DM** (n = 24)	**DN Macro** (n = 24)	**DN Micro** (n = 24)	**NP** (n = 24)	**p**
Age (years)	61.7 +/- 13.7	59.5 +/- 16.3	56.0 +/- 18.6	60.8 +/- 15.4	0.64
Sex					0.81
Male	9 (38%)	11 (45%)	12 (50%)	9 (39%)	
Female	15 (62%)	13 (55%)	12 (50%)	15 (61%)	
BMI	28.8 +/- 4.3	32.2 +/- 4.9	29.1 +/- 4.7	n.a.	0.12
Diabetes type					
1	6 (26%)	3 (14%)	10 (42%)	0 (0%)	0.11*
2	18 (74%)	21 (86%)	14 (58%)	0 (0%)	
none	0 (0%)	0 (0%)	0 (0%)	24 (100%)	
Arterial hypertensio	11 (46%)	14 (58%)	13 (54%)	1 (4%)	0.93
Systolic BP (mmHg)	133.6 +/- 12.8	149.1 +/- 16.2	140.2 +/- 16.0	n.a.	0.06
Diastolic BP (mmHg)	75.4 +/- 11.2	81.5 +/- 17.9	78.9 +/- 11.2	n.a.	0.53
ACE inhibitor	8 (33%)	12 (50%)	9 (38%)	1 (4%)	0.36
AT1 inhibitor	4 (17%)	4 (17%)	4 (17%)	0 (0%)	0.94
Renin	0 (0%)	1 (4%)	0 (0%)	0 (0%)	0.65
Ca Antagonist	4 (17%)	8 (33%)	4 (17%)	0 (0%)	0.33
Diuretics	4 (17%)	6 (26%)	7 (29%)	1 (4%)	0.41
Beta blocker	7 (29%)	6 (26%)	8 (33%)	1 (4%)	0.70
Antihypertensives	5 (21%)	6 (26%)	2 (8%)	n.a.	0.22

BMI: Body mass index

BP: Blood pressure

Supplemental Table 3 Protein identification, the four differentially excreted proteins between the investigated groups. The proteins were identified using mass spectrometry microsequencing and database comparisons. The accession numbers in Swiss-Prot, protein name, protein mass, pI, number of unique sequenced peptides and the sequence coverage are given.

Gene name	Swiss-Prot ID	Protein name	Masse (Da)	PI	Unique peptide sequenced	% Coverage
CDH1	CADH1_HUMAN	E-cadherin	97456.15	4.6	9	14
APOA1	APOA1_HUMAN	Apolipoprotein A-I	30758.93	5.6	18	56
REG1A	REG1A_HUMAN	Lithostathine-1-alpha	18730.98	5.6	7	55
B2M	B2MG_HUMAN	Beta-2-microglobulin	13714.57	6.1	4	26

Supplemental Table 4 Pairwise group comparisons of significant parameters in the complete study population. Bold p-values are considered significant at an unadjusted significance level 0.05 (RR systolic) or at a Bonferroni-adjusted significance level of 0.05/6=0.0083 (other parameters).

Parameter	Comparison	p
Systolic BP (mmHg)	DM versus DN Macro	**0.0086**
	DM versus DN Micro	0.8725
	DN Macro versus DN Micro	**0.0131**
BUN (mg/dl)	DM versus DN Macro	0.7210
	DM versus DN Micro	0.1047
	DM versus NP	0.1211
	DN Macro versus DN Micro	0.0117
	DN Macro versus NP	0.1100
	DN Micro versus NP	0.5462
Albuminuria (mg/l)	DM versus DN Macro	**0.0008**
	DM versus DN Micro	**< 0.0001**
	DM versus NP	0.1308
	DN Macro versus DN Micro	0.0372
	DN Macro versus NP	0.0769
	DN Micro versus NP	**< 0.0001**
APOA1	DM versus DN Macro	**0.0007**
	DM versus DN Micro	**< 0.0001**
	DM versus NP	**< 0.0001**
	DN Macro versus DN Micro	0.2812
	DN Macro versus NP	**< 0.0001**
	DN Micro versus NP	**< 0.0001**
B2M	DM versus DN Macro	**< 0.0001**
	DM versus DN Micro	**< 0.0001**
	DM versus NP	**< 0.0001**
	DN Macro versus DN Micro	0.8401
	DN Macro versus NP	**< 0.0001**
	DN Micro versus NP	**< 0.0001**
CDH1	DM versus DN Macro	**< 0.0001**
	DM versus DN Micro	**< 0.0001**
	DM versus NP	**< 0.0001**
	DN Macro versus DN Micro	**0.0033**
	DN Macro versus NP	**< 0.0001**
	DN Micro versus NP	**< 0.0001**
REG1A	DM versus DN Macro	**0.0001**
	DM versus DN Micro	**0.0015**
	DM versus NP	

	DN Macro versus DN Micro	**< 0.0001**
	DN Macro versus NP	0.3559
	DN Micro versus NP	**< 0.0001**
		**< 0.0001**

BP: Blood pressure

Supplemental Table 5 Pairwise group comparisons of significant parameters in the subpopulation with Western blot analysis. Bold p-values are considered significant at a Bonferroni-adjusted significance level of 0.05/6=0.0083.

**Parameter**	**Comparison**	**p**
Albuminuria (mg/l)	DM versus DN Macro	**0.0008**
	DM versus DN Micro	**< 0.0001**
	DM versus NP	0.1308
	DN Macro versus DN Micro	0.0372
	DN Macro versus NP	0.0769
	DN Micro versus NP	**< 0.0001**
APOA1	DM versus DN Macro	**0.0007**
	DM versus DN Micro	**< 0.0001**
	DM versus NP	**< 0.0001**
	DN Macro versus DN Micro	0.2812
	DN Macro versus NP	**< 0.0001**
	DN Micro versus NP	**< 0.0001**
B2M	DM versus DN Macro	**< 0.0001**
	DM versus DN Micro	**< 0.0001**
	DM versus NP	**< 0.0001**
	DN Macro versus DN Micro	0.8401
	DN Macro versus NP	**< 0.0001**
	DN Micro versus NP	**< 0.0001**
CDH1	DM versus DN Macro	**< 0.0001**
	DM versus DN Micro	**< 0.0001**
	DM versus NP	**< 0.0001**
	DN Macro versus DN Micro	**0.0033**
	DN Macro versus NP	**< 0.0001**
	DN Micro versus NP	**< 0.0001**
REG1A	DM versus DN Macro	**0.0001**
	DM versus DN Micro	**0.0015**
	DM versus NP	**< 0.0001**
	DN Macro versus DN Micro	0.3559
	DN Macro versus NP	**< 0.0001**
	DN Micro versus NP	**< 0.0001**

Supplemental Table 6 Results of ROC curve analyses to distinguish between two of the study groups using different parameters. Results are based on the complete study collective and Dot blot analysis.

Parameter	Comparison	Youden-optimal cutoff	Sensitivity %	Specificity %	AUC [95%-CI]
Systolic BP (mmHg)	DM vs DN Macro	150	59	84	0.74 [0.57, 0.90]
	DN Micro vs DN Macro	150	59	89	0.72 [0.54, 0.89]
Albuminuria	DM vs DN Macro	22	40	98	0.67 [0.57, 0.76]
	DM vs DN Micro	19	76	94	0.88 [0.81, 0.94]
	NP vs DN Micro	19	76	90	0.84 [0.75, 0.92]
APOA1	DM vs DN Macro	0.58	78	62	0.69 [0.60, 0.79]
	DM vs DN Micro	0.59	85	67	0.78 [0.69, 0.86]
	NP vs DM	0.29	98	100	0.98 [0.95, 1.00]
	NP vs DN Macro	0.29	100	100	1.00 [1.00, 1.00]
	NP bs DN Micro	0.29	97	100	0.97 [0.95, 1.00]
B2M	DM vs DN Macro	0.67	72	77	0.79 [0.75, 0.89]
	DM vs DN Micro	0.67	77	77	0.81 [0.75, 0.90]
	NP vs DM	0.37	100	94	0.99 [0.97, 1.00]
	NP vs DN Macro	0.49	97	97	0.97 [0.95, 1.00]
	NP vs DN Micro	0.49	98	97	0.98 [0.97, 1.00]
CDH1	DM vs DN Macro	1.01	47	97	0.74 [0.65, 0.83]
	DM vs DN Micro	1.01	63	97	0.85 [0.78, 0.92]
	NP vs DM	0.35	100	100	1.00 [1.00, 1.00]
	DN Macro vs DN Micro	1.19	37	88	0.64 [0.54, 0.74]
	NP vs DN Macro	0.35	100	100	1.00 [1.00, 1.00]
	NP vs DN Micro	0.35	100	100	1.00 [1.00, 1.00]
REG1A	DM vs DN Macro	0.42	38	92	0.67 [0.57, 0.77]
	DM vs DN Micro	0.35	57	78	0.67 [0.57, 0.77]
	NP vs DM	0.21	100	100	1.00 [1.00, 1.00]
	NP vs DN Macro	0.21	100	100	1.00 [1.00, 1.00]
	NP vs DN Micro	0.21	100	100	1.00 [1.00, 1.00]

BP: Blood pressure

Supplemental Table 7 Results of ROC curve analyses to distinguish between two of the study groups using different parameters. Results are based on the subpopulation with Western blot analysis.

**Parameter**	**Comparison**	**Youden-optimal cutoff**	**Sensitivity %**	**Specificity %**	**AUC** [95%-CI]
Albuminuria	DM vs DN Macro	18	74	100	0.89 [0.79, 1.00]
	DM vs DN Micro	16	79	96	0.89 [0.80, 0.99]
	NP vs DN Micro	19	75	90	0.84 [0.72, 0.96]
APOA1	DM vs DN Macro	2.77	100	100	1.00 [1.00, 1.00]
	DM vs DN Micro	2.26	88	96	0.94 [0.86, 1.00]
	NP vs DM	0.96	96	100	0.96 [0.89, 1.00]
	NP vs DN Macro	0.96	100	100	1.00 [1.00, 1.00]
	NP bs DN Micro	0.96	100	100	1.00 [1.00, 1.00]
B2M	DM vs DN Macro	1.02	96	58	0.72 [0.57, 0.87]
	DM vs DN Micro	3.30	46	83	0.62 [0.46, 0.79]
	NP vs DM	0.29	88	42	0.55 [0.38, 0.72]
	NP vs DN Macro	0.89	96	71	0.74 [0.57, 0.90]
	NP vs DN Micro	0.11	100	25	0.61 [0.45, 0.77]
CDH1	DM vs DN Macro	2.31	75	100	0.90 [0.80, 0.99]
	DM vs DN Micro	1.16	83	62	0.72 [0.57, 0.87]
	NP vs DM	0.36	83	100	0.90 [0.79, 1.00]
	DN Macro vs DN	1.94	79	88	0.85 [0.72, 0.97]
	Micro	0.36	100	100	100 [1.00, 1.00]
	NP vs DN Macro	0.36	100	100	100 [1.00, 1.00]
	NP vs DN Micro				
REG1A	DM vs DN Macro	0.16	50	83	0.69 [0.54, 0.84]
	DM vs DN Micro	0.16	38	83	0.54 [0.37, 0.71]
	NP vs DM	0.03	92	42	0.55 [0.38, 0.72]
	NP vs DN Macro	0.03	100	42	0.72 [0.57, 0.86]
	NP vs DN Micro	0.03	100	38	0.65 [0.49, 0.82]

References supplementary Materials and Methods

[1] Zhang Y, Bilbao A, Bruderer T, Luban J, Strambio-De-Castillia C, Lisacek F, Hopfgartner G, Varesio E (2015) The Use of Variable Q1 Isolation Windows Improves Selectivity in LC-SWATH-MS Acquisition. J Proteome Res. 14, 4359-71.

[2] Lambert, J. P., Ivosev, G., Couzens, A. L., Larsen, B. et al. (2013) Mapping differential interactomes by affinity purification coupled with data-independent mass spectrometry acquisition. Nat. Methods 10, 1239–1245.

[3] Tyanova, S., Temu, T., Sinitcyn, P., Carlson, A., Hein, M.Y., Geiger, T., Mann, M., Cox, J. (2016) The Perseus computational platform for comprehensive analysis of (prote)omics data. Nat Methods. 13, 731-40.

[4] Schmid H., Boucherot A., Yasuda Y., Henger A., Brunner B., Eichinger F., Nitsche A., Kiss E., Bleich M., Gröne H.J., Nelson P.J., Schlöndorff D., Cohen C.D., Kretzler M.; European Renal cDNA Bank (ERCB)

Consortium (2006). Modular activation of nuclear factor-kappaB transcriptional programs in human diabetic nephropathy. Diabetes. 55, 2993-3003.

[5] Ju W., Nair V., Smith S., Zhu L., Shedden K., et al. (2015) Tissue transcriptome driven identification of epidermal growth factor as a chronic kidney disease biomarker. Sci. Transl. Med. 2; 7(316):
